# Cognitive Frailty and Health Care Utilization and Costs Among Middle-Aged and Older Adults: Findings from Four Longitudinal Cohort Studies

**DOI:** 10.14336/AD.2025.0722

**Published:** 2026-07-31

**Authors:** Yemin Yuan, Zhenyu Shi, Xiaolong Guan, Yiqi Xia, Yanshang Wang, Huaxin Si, Ping He

**Affiliations:** ^1^School of Public Health, Peking University, Beijing, 100191, China.; ^2^China Center for Health Development Studies, Peking University, Beijing, 100191, China.

**Keywords:** frailty, cognitive impairment, healthcare utilization, expenditures

## Abstract

Health care systems need better strategies to identify older adults at risk for costly care to select target populations for interventions to reduce health care burden. Cognitive impairment and frailty are among the two most common geriatric syndromes. We examined the association between cognitive frailty and health care utilization and costs. Participants aged 50 and over were drawn from four prospective cohorts of aging, including the China Health and Retirement Longitudinal Study (CHARLS), the Korean Longitudinal Study of Aging (KLoSA), the Mexican Health and Aging Study (MHAS), and the Survey of Health, Ageing and Retirement in Europe (SHARE). We classified participants according to their cognitive impairment and frailty status into the following groups: none, only cognitive impairment, only frailty and cognitive frailty. We used negative binomial regression models and sample selection models to explore the association between cognitive frailty and health care utilization and costs. Compared to participants without cognitive impairment and frailty, participants in the only frailty or cognitive frailty groups had higher average annual outpatient visits and inpatient admissions. Only cognitive impairment was significantly negatively associated with the probability of outpatient visits or out-of-pocket (OOP) costs. Only frailty was significantly associated with a higher probability of outpatient visits or more OOP costs. The association between cognitive frailty and outpatient visits varied by cohort. In KLoSA, cognitive frailty was associated with a lower probability of outpatient visits, whereas the other three cohorts show the opposite. Cognitive frailty was associated with higher outpatient costs in CHARLS, KLoSA and MHAS. Only frailty and cognitive frailty were associated with a higher likelihood of inpatient admission in each cohort, and they were also linked to higher inpatient costs in both KLoSA and SHARE. But cognitive frailty was associated with lower inpatient costs in MHAS. This study highlights the significant positive association between cognitive frailty and health care utilization and OOP costs in most countries. By recognizing the multifaceted nature of cognitive impairment and frailty, healthcare providers and policymakers can work towards more effective interventions and support systems that address the needs of middle-aged and older adults, ultimately improving their quality of life and reducing health care costs.

## INTRODUCTION

By 2050, the world’s population of people aged 60 years and older will double (2.1 billion) (www.who.int/news-room/fact-sheets/detail/ageing-and-health). All countries face major challenges to ensure that their health systems are ready to make the most of this demographic shift. The World Health Organization report shows that global spending on health continually rose between 2000 and 2018 and reached US$ 8.3 trillion or 10% of global GDP (www.who.int/publications/i/item/9789240017788). Spending on health is outpacing the rest of the global economy (news.un.org/en/story/2019/02/1033191). It is important to identify the specific elements that may increase the cost of spending.

Cognitive impairment (CI) and frailty are among the two most common geriatric syndromes [1]. Mild cognitive impairment (MCI) is the stage between the expected cognitive decline of normal aging and the more serious decline of dementia [2]. The overall conversion rate from MCI to dementia was 35% [3]. In 2019, the global cost of dementia was estimated to be US$ 1.3 trillion (www.who.int/news-room/fact-sheets/detail/dementia). Several studies have investigated the magnitude of the CI-related burden on health care utilization and medical expenditures [4, 5]. The prevalence of frailty is rapidly increasing globally along with population ageing and will increase health-care demand [6]. Compared to those without frailty (£957.27), the total annual costs of individual primary and secondary care doubled in mild frailty (£2,158.95), tripled in moderate (£3,219.83) and more than quadrupled in severe frailty (£4,464.23) [7].

In 2013, an international consensus group proposed the concept and operational definition of cognitive frailty; that is, co-occurrence of cognitive impairment and frailty without concurrent Alzheimer’s disease or dementia [8]. Research demonstrated that 20% of people with frailty also had cognitive impairment [9]. A recent review study reported a median prevalence of cognitive frailty among community-dwelling older adults of 12.2% [10]. Despite the high prevalence of cognitive frailty in the population, there are several key gaps in knowledge about its effects on outcomes such as healthcare utilization and costs [11, 12]. Cognitive frailty is a risk factor for adverse outcomes (e.g., falls, disability, hospitalization, dementia, and all-cause mortality) independent of comorbidity, health behaviors, and mental health [12, 13]. These impairments may also adversely affect a person’s capacity to self-manage chronic conditions and access preventive care, which could lead to higher utilization of health care and spiraling cost. Understanding how cognitive frailty influences healthcare needs is crucial for developing effective management strategies and allocating resources efficiently. But there has been little research on the impact of cognitive frailty on health care. In addition, the relationship between cognitive frailty and health care utilization and costs would vary due to differences in national health systems.

To fill these research gaps, we conducted a cross-cultural, longitudinal analysis based on four large, comparative cohort studies between 2010 and 2018 that represented adults aged 50 years and older from three continents - North America, Europe, and Asia. This study aimed to examine the association between cognitive frailty and health care utilization and costs.

## MATERIALS AND METHODS

### Study design and participants

Data were obtained from four international cohorts of ageing: the China Health and Retirement Longitudinal Study (CHARLS) [14], the Korean Longitudinal Study of Aging (KLoSA) [15], the Mexican Health and Aging Study (MHAS) [16], and the Survey of Health, Ageing and Retirement in Europe (SHARE) [17]. The four surveys used here were designed to provide comparable results. In this study, we use data from a similar time range: 2011-2018 for CHARLS, 2010-2018 for KLoSA, 2012-2018 for MHAS, 2013-2017 for SHARE (Supplementary Table 1). We excluded participants who were younger than 50 years of age and who reported to have been diagnosed with dementia or Parkinson’s disease by a doctor. The reasons for selecting individuals aged 50 and over include the following: this age range represents a critical period where cognitive decline and frailty begin to significantly emerge and progress [18]; these conditions pose an increasing burden on healthcare systems within this age group [7]; and the international cohort studies utilized are specifically designed to track the aging process within this demographic. Participants were also excluded if they had missing information on cognitive function, frailty, health care utilization and expenditure or covariates. After excluding these participants, 36,272 participants from CHARLS, 30,816 participants from KLoSA, 40,872 participants from MHAS, and 92,265 participants from SHARE (Austria, Belgium, Croatia, Czech Republic, Denmark, Estonia, France, Germany, Greece, Israel, Italy, Luxembourg, Netherlands, Poland, Portugal, Slovenia, Spain, Sweden, and Switzerland) were available for the analysis (Supplementary Fig. 1).

### Measurements

The same measurements of exposure (cognitive frailty), outcome (health care utilization and costs), and covariates are used in each cohort.

Three sets of cognitive function tests were completed to assess cognitive impairment, including an immediate word recall test, a delayed word recall test, and an orientation to date test (Supplementary Table 2). These primarily assessed the cognitive domains of memory and orientation. Participants who scored 1.5 standard deviations below the mean in two or three tests, compared to the total population aged 50 and over with the same level of education within the database were defined as cognitive impairment [19, 20]. Frailty was evaluated by the frailty index (FI). Following the deficit accumulation model developed by Searle et al. [21], we constructed the FI using a standard procedure (Supplementary Table 3). To harmonize the frailty assessment of data from the four cohorts, we selected 29 items to construct the FI, including variables of self-reported health status, functional limitations, mobility, chronic diseases, and psychological characteristics. We excluded participants that they had >20% missing data in items of frailty index. FI was calculated as the sum of scoring divided by the total number of items. Frailty status was defined as a value of 0.25 or greater on the FI [22]. We further classified participants according to their cognitive impairment and frailty status into the following groups: none (without cognitive impairment and frailty), only cognitive impairment, only frailty, and cognitive frailty (co-occurrence of cognitive impairment and frailty) [8].

Health care utilization included the annual number of outpatient visits per capita and the annual number of inpatient admissions per capita. Health care costs were measured by the average out-of-pocket (OOP) costs per visit. Due to significant variations in national health systems (e.g., public healthcare coverage, insurance reimbursement rates), focusing solely on OOP costs may not fully reflect the total healthcare burden. In CHARLS, the time span of outpatient utilization and expenditures was the past month, and the time span of inpatient utilization and expenditures was the past year. In KLoSA, the time span for both outpatient and inpatient utilization and costs was the last two years. In both MHAS and SHARE, the time span of outpatient and inpatient utilization and costs was within the past year. See Supplementary Table 4 for details. For a better understanding, all outcome variables were uniformly converted to one-year time units [23]. Health care costs were adjusted for inflation using the consumer price index for each country. We report adjusted 2018 dollar amounts for each cohort.

Covariates were identified through literature reviews. We included age (in years), sex (male, female), education (less than upper secondary, upper secondary and vocational training, and tertiary), residence (urban, rural), household wealth (low tertile, medium tertile, and high tertile), marital status (single, married or partnered), smoking (no, yes), alcohol drinking (no, yes), and public health insurance (no, yes). Further details about covariates are available in Supplementary Methods.

### Statistical analysis

Median with interquartile range (IQR) was used for reporting descriptive statistics of continuous variables, and number with percentage was used for reporting categorical variables. Differences in sociodemographic, risk factors, and insurance status across the four categories (none, only cognitive impairment, only frailty, and cognitive frailty) were assessed using Kruskal-Wallis test for continuous but non-normally distributed variables and chi-square test for categorical variables. Mean with standard deviation (SD) [24] was used for reporting healthcare utilization and costs across the four categories. The Kruskal-Wallis Test was used to compare differences between the four groups. The control group were participants without cognitive impairment and frailty.

We used negative binomial regression models to analyze the association between cognitive frailty and health care utilization as count data because the variances were greater than the means in outcome variables [25] (Supplementary Table 5). This indicates that excessive overdispersion was present in the outcome variables. As cognitive frailty status gradually progresses over time, we used mixed models to account for the longitudinal association between cognitive frailty and health care utilization (binary) and costs [26, 27]. Mixed models took into account the within-subject correlation of time-varying cognitive frailty status and healthcare utilization during follow-up. We used sample selection models to explore the association between cognitive frailty and health care utilization (binary) and costs. The first step was the selection model, and the mixed logistic regression model was used to estimate the probability of using health care. The second step was to estimate the direct costs by a mixed linear regression model on log (positive) expenditures. The commands for the mixed logistic regression model and the mixed linear regression model in Stata are “melogit” and “mixed” respectively. We controlled for individual-level effects using "|| ID:". All variances and covariances are distinctly estimated. More details on the introduction of the sample selection models can be found in Supplementary Methods. We also supplemented the analysis by calculating the total aggregated annual OOP expenses for each participant, encompassing outpatient visits and inpatient admissions.

Several sensitivity analyses were conducted. Firstly, we further conducted our analysis based on inverse probability-weighted samples to handle potential selection bias. Secondly, we used two-part models to explore the association between cognitive frailty and health care utilization and costs. In the first part, the logit model was used to estimate the probability. In the second part, the generalized linear model (GLM) with a gamma distribution and a log link was used to assess the association of average OOP costs with cognitive frailty. More information on the introduction of the two-part models and the appropriateness of specifying the model using gamma distribution and log link was provided in Supplementary Methods. Thirdly, we reconstructed the FI based on each of the four studies (Supplementary Table 6). All analyses were performed by Stata 15.0 (StataCorp, College Station, USA). All hypothesis tests were two-sided with a significance level of *p*<0.05.

## RESULTS

### Sample characteristics

**Table 1:** Summary statistics of study sample by cognitive frailty.

Characteristics	CHARLS (n=36,272)					KLoSA (n=30,816)				
	None (n=24,388)	Only cognitive impairment (n=3,032)	Only frailty (n=7,007)	Cognitive frailty (n=1,845)	*P* value	None (n=25,421)	Only cognitive impairment (n=3,202)	Only frailty (n=1,045)	Cognitive frailty (n=1,148)	*P* value
Age (years), median (IQR)	60 (54-65)	64 (58-71)	63 (57-69)	68 (61-75)	<0.001	66 (59-73)	73 (64-80)	77 (71-83)	82 (76-88)	<0.001
Female, n (%)	10,678 (43.8)	1,601 (52.8)	4,328 (61.8)	1,271 (68.9)	<0.001	14,851 (58.4)	1,891 (59.1)	655 (62.7)	754 (65.7)	<0.001
Education, n (%)					<0.001					<0.001
Less than upper secondary	20,986 (86.1)	2,658 (87.7)	6,589 (94.0)	1,744 (94.5)		15,383 (60.5)	1,739 (54.3)	918 (87.8)	922 (80.3)	
Upper secondary and vocational training	2,989 (12.3)	329 (10.9)	378 (5.4)	85 (4.6)		7,539 (29.7)	1,184 (37.0)	101 (9.7)	171 (14.9)	
Tertiary	413 (1.7)	45 (1.5)	40 (0.6)	16 (0.9)		2,499 (9.8)	279 (8.7)	26 (2.5)	55 (4.8)	
Residence, n (%)					<0.001					<0.001
Urban	10,157 (41.6)	842 (27.8)	2,407 (34.4)	464 (25.1)		18,957 (74.6)	2,268 (70.8)	753 (72.1)	824 (71.8)	
Rural	14,231 (58.4)	2,190 (72.2)	4,600 (65.6)	1,381 (74.9)		6,464 (25.4)	934 (29.2)	292 (27.9)	324 (28.2)	
Household wealth, n (%)					<0.001					<0.001
Low tertile	7,555 (31.0)	1,264 (41.7)	2,889 (41.2)	970 (52.6)		10,061 (39.6)	1,507 (47.1)	549 (52.5)	735 (64.0)	
Medium tertile	7,409 (30.4)	1,052 (34.7)	2,440 (34.8)	605 (32.8)		6,562 (25.8)	757 (23.6)	268 (25.6)	242 (21.1)	
High tertile	9,424 (38.6)	716 (23.6)	1,678 (23.9)	270 (14.6)		8,798 (34.6)	938 (29.3)	228 (21.8)	171 (14.9)	
Married or partnered, n (%)	21,664 (88.8)	2,444 (80.6)	5,746 (82.0)	1,308 (70.9)	<0.001	19,697 (77.5)	2,049 (64.0)	623 (59.6)	511 (44.5)	<0.001
Smoking, n (%)	11,745 (48.2)	1,329 (43.8)	2,711 (38.7)	598 (32.4)	<0.001	7,621 (30.0)	921 (28.8)	340 (32.5)	311 (27.1)	0.021
Alcohol drinking, n (%)	12,031 (49.3)	1,301 (42.9)	2,765 (39.5)	680 (36.9)	<0.001	24,130 (94.9)	3,040 (94.9)	955 (91.4)	1,048 (91.3)	<0.001
Public health insurance, n (%)	23,150 (94.9)	2,818 (92.9)	6,606 (94.3)	1,698 (92.0)	<0.001	24,452 (96.2)	2,985 (93.2)	912 (87.3)	1,004 (87.5)	<0.001

Characteristics	MHAS (n=40,872)					SHARE (n=92,265)				
	None (n=24,971)	Only cognitive impairment (n=3,358)	Only frailty (n=9,894)	Cognitive frailty (n=2,649)	*P* value	None (n=63,077)	Only cognitive impairment (n=5,150)	Only frailty (n=19,319)	Cognitive frailty (n=4,719)	*P* value

Age (years), median (IQR)	62 (56-69)	70 (62-78)	67 (60-74)	77 (69-83)	<0.001	64 (58-71)	72 (63-79)	71 (63-78)	79 (72-85)	<0.001
Female, n (%)	13,340 (53.4)	1,265 (37.7)	7,279 (73.6)	1,577 (59.5)	<0.001	33,197 (52.6)	2,184 (42.4)	13,088 (67.7)	2,866 (60.7)	<0.001
Education, n (%)					<0.001					<0.001
Less than upper secondary	20,106 (80.5)	2,768 (82.4)	9,268 (93.7)	2,487 (93.9)		22,536 (35.7)	1,687 (32.8)	10,297 (53.3)	2,645 (56.1)	
Upper secondary and vocational training	1,034 (4.1)	120 (3.6)	168 (1.7)	42 (1.6)		24,327 (38.6)	1,960 (38.1)	6,400 (33.1)	1,387 (29.4)	
Tertiary	3,831 (15.3)	470 (14.0)	458 (4.6)	120 (4.5)		16,214 (25.7)	1,503 (29.2)	2,622 (13.6)	687 (14.6)	
Residence, n (%)					<0.001					<0.001
Urban	18,039 (72.2)	1,980 (59.0)	7,008 (70.8)	1,637 (61.8)		43,203 (68.5)	3,597 (69.8)	12,749 (66.0)	3,247 (68.8)	
Rural	6,932 (27.8)	1,378 (41.0)	2,886 (29.2)	1,012 (38.2)		19,874 (31.5)	1,553 (30.2)	6,570 (34.0)	1,472 (31.2)	
Household wealth, n (%)					<0.001					<0.001
Low tertile	8,929 (35.8)	1,456 (43.4)	4,148 (41.9)	1,252 (47.3)		18,874 (29.9)	1,729 (33.6)	8,027 (41.5)	2,129 (45.1)	
Medium tertile	7,358 (29.5)	901 (26.8)	2,972 (30.0)	745 (28.1)		20,802 (33.0)	1,775 (34.5)	6,820 (35.3)	1,651 (35.0)	
High tertile	8,684 (34.8)	1,001 (29.8)	2,774 (28.0)	652 (24.6)		23,401 (37.1)	1,646 (32.0)	4,472 (23.1)	939 (19.9)	
Married or partnered, n (%)	18,190 (72.8)	2,259 (67.3)	6,117 (61.8)	1,322 (49.9)	<0.001	50,772 (80.5)	3,789 (73.6)	12,718 (65.8)	2,648 (56.1)	<0.001
Smoking, n (%)	9,974 (39.9)	1,403 (41.8)	3,393 (34.3)	968 (36.5)	<0.001	30,342 (48.1)	2,432 (47.2)	8,778 (45.4)	1,798 (38.1)	<0.001
Alcohol drinking, n (%)	7,293 (29.2)	919 (27.4)	1,606 (16.2)	337 (12.7)	<0.001	37,452 (59.4)	2,627 (51.0)	8,049 (41.7)	1,436 (30.4)	<0.001
Public health insurance, n (%)	21,863 (87.6)	2,843 (84.7)	9,067 (91.6)	2,324 (87.7)	<0.001	-	-	-	-	-

CHARLS: China Health and Retirement Longitudinal Study; KLoSA: Korean Longitudinal Study of Aging; MHAS: Mexican Health and Aging Study; SHARE: Survey of Health, Ageing and Retirement in Europe; IQR: interquartile range.

### Annual utilization of health care and associated costs

**Figure 1.**
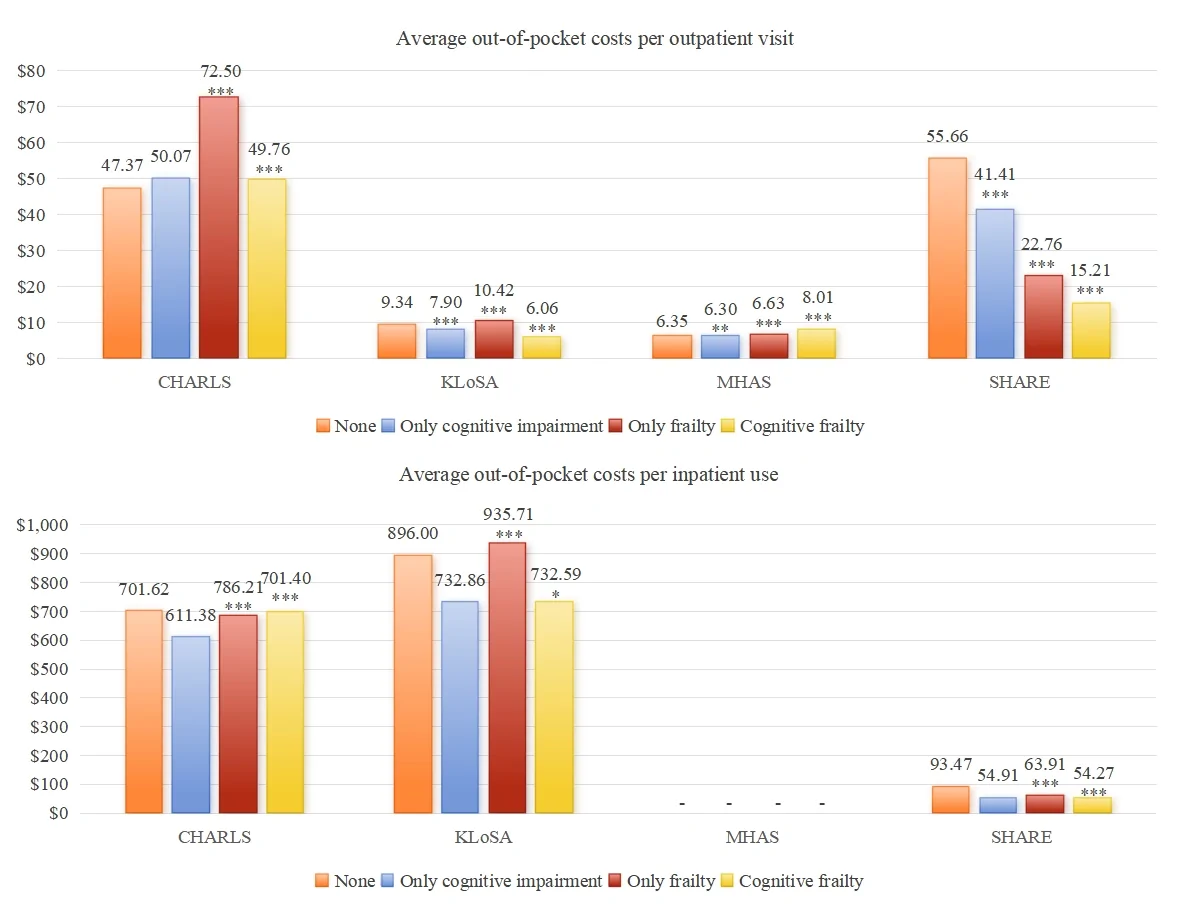
**Average out-of-pocket costs per health care visit**. The control group were participants without cognitive impairment and frailty. ***p<0.001, **p<0.01, *p<0.05. We report adjusted 2018 dollar amounts for each cohort.

An overview of outpatient visits and inpatient admissions, and their associated costs, are presented in Table 2 and Figure 1. Regarding the relevant costs, we report adjusted 2018 dollar amounts for each cohort. Across all four cohorts, participants in the only frailty and cognitive frailty groups had higher average annual outpatient visits than participants in the no cognitive impairment and frailty groups. In CHARLS and MHAS, the participants with only frailty and cognitive frailty had higher average annual outpatient OOP costs than those without cognitive impairment and frailty, while participants who have only frailty and cognitive frailty have lower costs in SHARE. Average OOP costs per outpatient visit displayed a similar pattern to average annual outpatient OOP costs. In CHARLS, KLoSA, and SHARE, compared to participants without cognitive impairment and frailty, participants classified as only frailty and cognitively frailty had higher average annual inpatient visits. Participants with only frailty and cognitive frailty had higher average annual inpatient OOP costs compared to those without cognitive impairment and frailty across all cohorts.

### Association between cognitive frailty on health care utilization and costs

As shown in Table 3, healthcare utilization was analyzed as count data. Only frailty or cognitive frailty was associated with a higher number of outpatient visits and inpatient admissions. Only cognitive impairment was negatively associated with the number of outpatient visits in KLoSA (*β*=-0.109, SE=0.022, *P*<0.001) and MHAS (*β*=-0.091, SE=0.023, *P*<0.001). Only cognitive impairment was positively associated with the number of outpatient visits (*β*=0.037, SE=0.015, *P*=0.010) and inpatient admissions (*β*=0.166, SE=0.044, *P*<0.001) in SHARE.

Table 4 reports the estimated parameters and features of the sample selection model for health care utilization (binary) and costs. Only frailty was associated with a higher probability of outpatient visits in CHARLS (*β*=0.805, SE=0.041, *P*<0.001), MHAS (*β*=0.941, SE=0.044, *P*<0.001), and SHARE (*β*=1.820, SE=0.073, *P*<0.001). The same is true for cognitive frailty (CHARLS: *β*=0.701, SE=0.072, *P*<0.001; MHAS: *β*=0.605, SE=0.073, *P*<0.001; SHARE: *β*=0.532, SE=0.093, *P*<0.001). But cognitive frailty was associated with a lower probability of outpatient visits in KLoSA (*β*=-0.427, SE=0.110, *P*<0.001). In addition, only cognitive impairment was negatively associated with the probability of outpatient visits in KLoSA (*β*=-0.193, SE=0.062, *P*=0.002), MHAS (*β*=-0.208, SE=0.054, *P*<0.001) and SHARE (*β*=-0.503, SE=0.069, *P*<0.001). In CHARLS, KLoSA, and MHAS, only frailty and cognitive frailty were positively associated with outpatient costs. Only cognitive impairment was negatively associated with outpatient costs in CHARLS and KLoSA. The visits probability or costs of outpatient of frailty alone were higher than those of cognitive frailty.

**Table 2:** Summary of health care utilization and associated cost.

	CHARLS (n=36,272)			KLoSA (n=30,816)	
	The average number of annual visits, mean (SD)	Average annual OOP costs, US dollars, mean (SD)		The average number of annual visits, mean (SD)	Average annual OOP costs, US dollars, mean (SD)
Outpatient					
None	3.68 (0.09)	174.32 (8.98)		6.57 (0.06)	61.34 (1.54)
Only cognitive impairment	3.38 (0.23)	169.22 (34.95)		6.87* (0.20)	54.27*** (3.07)
Only frailty	7.94*** (0.25)	575.68*** (53.52)		10.53*** (0.39)	109.67 (12.72)
Cognitive frailty	7.35*** (0.44)	365.77*** (52.85)		9.86*** (0.46)	59.75*** (5.94)
Inpatient					
None	0.13 (0.00)	91.21 (4.14)		0.06 (0.00)	53.76 (2.62)
Only cognitive impairment	0.13 (0.01)	79.48 (10.63)		0.07 (0.00)	51.30 (6.80)
Only frailty	0.39*** (0.01)	267.62*** (14.69)		0.21*** (0.01)	196.50*** (34.24)
Cognitive frailty	0.35*** (0.02)	245.49*** (32.57)		0.22*** (0.01)	161.17* (25.27)

	MHAS (n=40,872)			SHARE (n=92,265)	
	The average number of annual visits, mean (SD)	Average annual OOP costs, US dollars, mean (SD)		The average number of annual visits, mean (SD)	Average annual OOP costs, US dollars, mean (SD)

Outpatient					
None	4.72 (0.04)	29.97 (1.04)		4.95 (0.03)	275.52 (2.75)
Only cognitive impairment	4.34*** (0.10)	27.34** (2.61)		5.38** (0.11)	221.56*** (8.89)
Only frailty	8.32*** (0.10)	55.13*** (2.70)		11.28*** (0.09)	256.72*** (4.58)
Cognitive frailty	7.50*** (0.19)	60.07*** (5.65)		11.39*** (0.21)	173.28*** (6.97)
Inpatient					
None	-	41.19 (2.73)		0.15 (0.00)	14.02 (0.94)
Only cognitive impairment	-	45.38 (7.51)		0.22** (0.01)	12.08 (2.10)
Only frailty	-	101.54*** (8.16)		0.53*** (0.01)	33.87*** (1.65)
Cognitive frailty	-	109.26*** (16.06)		0.62*** (0.02)	33.65*** (3.96)

CHARLS: China Health and Retirement Longitudinal Study; KLoSA: Korean Longitudinal Study of Aging; MHAS: Mexican Health and Aging Study; SHARE: Survey of Health, Ageing and Retirement in Europe; OOP: out-of-pocket; SD: Standard deviation. Regarding the relevant costs, we report adjusted 2018 dollar amounts for each cohort. The control group were participants without cognitive impairment and frailty. ***p<0.001, **p<0.01, *p<0.05.

Only frailty and cognitive frailty were associated with a higher probability of inpatient in the four cohorts, and they were associated with higher inpatient costs in KLoSA and SHARE. Only frailty was associated with higher inpatient costs in CHARLS (*β*=0.177, SE=0.047, *P*<0.001). In MHAS, only cognitive impairment (*β*=-0.413, SE=0.187, *P*=0.027) and cognitive frailty (*β*=-0.457, SE=0.157, *P*=0.004) were associated with lower inpatient costs. The inpatient probability of frailty alone was higher than that of cognitive frailty in CHARLS, while the inpatient probability of cognitive frailty in KLoSA, MHAS, and SHARE was higher. The inpatient costs of cognitive frailty were higher than those of frailty alone in KLoSA and SHARE. The results of cognitive frailty on health care utilization and total aggregated annual OOP costs are presented in Supplementary Table 7. Only cognitive impairment was negatively associated with the probability of healthcare use in KLoSA, MHAS and SHARE. Cognitive frailty was positively associated with the healthcare utilization and OOP costs in most countries.

In sensitivity analyses, we conducted our analysis based on inverse probability-weighted samples. In KLoSA, the inpatient probability of cognitive frailty was higher than that of frailty alone. The other results are basically consistent with the existing results (Supplementary Table 8). For the results of the two-part models, the utilization of health care was similar to the present, but the coefficient of health care costs was not significant in MHAS and SHARE (Supplementary Table 9). The results after reassessment of the FI were generally consistent with existing findings (Supplementary Table 10).

## Discussion

Our study analyzed data from 200,225 individuals aged 50 years and older from four cohorts on ageing to explore the association of cognitive frailty with health care utilization and costs. Cognitive impairment alone was negatively associated with health care utilization or annual OOP costs. Frailty alone was positively associated with utilization or annual OOP costs. However, the association between cognitive frailty and health care utilization or annual OOP costs varies depending on service type and cohort.

We found that the presence of cognitive impairment alone was generally associated with reduced health care utilization or annual OOP costs. This is similar to previous findings. Among adults aged and over 65 in the United Kingdom, those with cognitive impairment are at significantly lower odds of using allied health services than those without [28]. Cognitive impairment is also a barrier to utilizing preventive services. Adults over 50 years of age with cognitive impairment in the United States were at risk for underutilizing preventive health services [29]. A qualitative study from China found that the main influencing factors of the help-seeking behavior in patients with mild cognitive impairment included perceived disease threat, symptom attribution, disease knowledge, use of cognitive compensation strategies, predictable burden, social support, economic conditions, and accessibility of medical services [30]. However, we found that only cognitive impairment was associated with increased use of health care in SHARE (Table 3). Universal healthcare systems in European countries often prioritize early diagnosis and intervention for early cognitive decline, which may explain the increased healthcare use [31]. It may also be influenced by reimbursement policies that encourage regular check-ups and early interventions [32]. Additionally, if impairments in other cognitive domains (e.g., executive function, attention) have different impacts on healthcare utilization, this study may not have fully captured them. The relationship between cognitive impairment and health care utilization and costs needs to be further verified in the future.

**Table 3:** Regression results of cognitive frailty on health care utilization as count data.

	CHARLS (n=36,272)		KLoSA (n=30,816)
Outpatient			
None	Ref		Ref
Only cognitive impairment	-0.119 (0.096)		-0.109*** (0.022)
Only frailty	0.748*** (0.068)		0.206*** (0.036)
Cognitive frailty	0.639*** (0.121)		0.049 (0.035)
Inpatient			
None	Ref		Ref
Only cognitive impairment	-0.060 (0.066)		0.062 (0.075)
Only frailty	1.051*** (0.038)		1.088*** (0.076)
Cognitive frailty	0.873*** (0.066)		1.122*** (0.076)

	**MHAS (n=40,872)**		**SHARE^a^ (n=92,265)**

Outpatient			
None	Ref		Ref
Only cognitive impairment	-0.091*** (0.023)		0.037* (0.015)
Only frailty	0.477*** (0.015)		0.792*** (0.008)
Cognitive frailty	0.340*** (0.026)		0.756*** (0.015)
Inpatient			
None	-		Ref
Only cognitive impairment	-		0.166*** (0.044)
Only frailty	-		1.169*** (0.023)
Cognitive frailty	-		1.182*** (0.039)

CHARLS: China Health and Retirement Longitudinal Study; KLoSA: Korean Longitudinal Study of Aging; MHAS: Mexican Health and Aging Study; SHARE: Survey of Health, Ageing and Retirement in Europe. Models were adjusted for: age, sex, education, residence, household wealth, marital status, smoking, alcohol drinking, public health insurance and survey year. Standard errors in parentheses.

a SHARE was not adjusted for insurance variables. ***P<0.001, **P<0.01, *P<0.05.

The observation that cognitive impairment is associated with reduced health care utilization could suggest that individuals with cognitive impairment may have less access to healthcare services [33], possibly due to factors like a lack of awareness of health needs, or barriers in communication. Alternatively, it could also imply that caregivers or family members may be less likely to seek out health care for individuals with cognitive impairments. The finding that cognitive impairment is linked to lower annual OOP costs might seem counterintuitive, as one might expect that individuals with cognitive impairments would incur higher costs due to increased health care needs. However, this could reflect a lower frequency of healthcare visits, fewer prescriptions, or a reliance on informal care rather than formal healthcare services. There is evidence that cognitive impairment was associated with higher home health care delivery costs [34]. Home health utilization begins to significantly increase in the years preceding a dementia diagnosis [35]. These may reduce institutional health care utilization and costs for people with cognitive impairment.

The total aggregated annual OOP expenses analysis further supports the “offsetting effect” of cognitive impairment alone. For participants with only cognitive impairment, the reduced probability of healthcare utilization directly leads to lower total annual OOP expenses, which aligns with the observed negative association between isolated cognitive impairment and healthcare costs. In contrast, for those with cognitive frailty, the “offsetting effect” is weakened or even reversed: the higher healthcare utilization driven by frailty outweighs the reduced utilization due to cognitive impairment alone, resulting in higher total aggregated annual OOP expenses in most cohorts. This combined perspective clarifies that the lower costs in cases of isolated cognitive impairment are primarily driven by reduced service use, while the higher costs in cognitive frailty reflect the dominant impact of frailty on increasing healthcare needs and associated expenditures.

Consistent with previous research, this study found that frailty alone was associated with higher health care utilization or OOP costs. A 0.1-increase in frailty was significantly associated with the total number of outpatient visits (IRR = 1.35) and inpatient (IRR = 1.60) admissions over time, and length of stay (IRR = 1.12) [36]. Frail individuals experienced an increased risk for outpatient and inpatient use compared to their robust counterpart [37, 38]. Moreover, frailty was associated with health care expenditure in a variety of contexts [39, 40], even after accounting for chronic conditions and disability [41]. Self-reported functional impairments and phenotypic frailty are also robustly associated with substantial additional health care costs in community-dwelling older adults [42].

**Table 4:** Regression results of cognitive frailty on health care utilization (binary) and costs.

	CHARLS (n=36,272)			KLoSA (n=30,816)	
	Selection model, whether it occurs	Costs model, ln (costs)		Selection model, whether it occurs	Costs model, ln (costs)
Outpatient					
None	Ref	Ref		Ref	Ref
Only cognitive impairment	-0.103 (0.066)	-0.170* (0.083)		-0.193** (0.062)	-0.060* (0.026)
Only frailty	0.805*** (0.041)	0.484*** (0.046)		0.102 (0.121)	0.438*** (0.046)
Cognitive frailty	0.701*** (0.072)	0.358*** (0.082)		-0.427*** (0.110)	0.260*** (0.050)
Inpatient					
None	Ref	Ref		Ref	Ref
Only cognitive impairment	-0.080 (0.076)	-0.069 (0.089)		-0.015 (0.072)	-0.064 (0.103)
Only frailty	1.172*** (0.046)	0.177*** (0.047)		1.283*** (0.092)	0.382** (0.118)
Cognitive frailty	0.983*** (0.077)	0.117 (0.079)		1.309*** (0.093)	0.680*** (0.132)

	MHAS (n=40,872)			SHARE^a^ (n=92,265)	
	Selection model, whether it occurs	Costs model, ln (costs)		Selection model, whether it occurs	Costs model, ln (costs)

Outpatient					
None	Ref	Ref		Ref	Ref
Only cognitive impairment	-0.208*** (0.054)	-0.095 (0.060)		-0.503*** (0.069)	0.010 (0.029)
Only frailty	0.941*** (0.044)	0.450*** (0.036)		1.820*** (0.073)	0.025 (0.016)
Cognitive frailty	0.605*** (0.073)	0.311*** (0.062)		0.532*** (0.093)	0.058 (0.034)
Inpatient					
None	Ref	Ref		Ref	Ref
Only cognitive impairment	0.099 (0.075)	-0.413* (0.187)		0.085 (0.051)	-0.104 (0.096)
Only frailty	1.233*** (0.043)	-0.110 (0.106)		1.336*** (0.029)	0.117** (0.044)
Cognitive frailty	1.312*** (0.067)	-0.457** (0.157)		1.393*** (0.046)	0.207** (0.076)

CHARLS: China Health and Retirement Longitudinal Study; KLoSA: Korean Longitudinal Study of Aging; MHAS: Mexican Health and Aging Study; SHARE: Survey of Health, Ageing and Retirement in Europe. Models were adjusted for: age, sex, education, residence, household wealth, marital status, smoking, alcohol drinking, public health insurance, and survey year. Standard errors in parentheses.

a SHARE was not adjusted for insurance variables. ***P<0.001, **P<0.01, *P<0.05.

Frailty is a clinical syndrome characterized by decreased physiological reserve and increased vulnerability to stressors, leading to a higher risk of adverse health outcomes [43]. Frail individuals tend to visit healthcare providers more frequently for management of chronic conditions and preventive care. They are also more likely to be hospitalized due to acute health issues, complications from chronic diseases, or falls. Frail individuals often incur higher OOP costs due to [40]: increased frequency of medical visits; higher prescription medication needs; potential costs associated with rehabilitation services following hospitalizations. Continued research is still necessary to explore the specific mechanisms through which frailty leads to increased healthcare utilization and costs.

We also found that cognitive frailty was associated with higher health care utilization and OOP costs. However, there were two exceptions. In KLoSA, cognitive frailty was associated with lower outpatient utilization; and in MHAS, cognitive frailty was associated with lower inpatient costs (Table 4). This difference may be influenced by the association between cognitive impairment alone and health care utilization or costs. In KLoSA’s selection model and MHAS’s costs model, only cognitive impairment had a negative association coefficient with outpatient utilization or inpatient costs, while frailty alone had no statistically significant association with outpatient service utilization or inpatient costs. In South Korea, strict referral systems and limited reimbursement for cognitive assessments in primary care could suppress outpatient utilization [44]. Cultural reliance on family care may reduce formal healthcare utilization for cognitive issues, whereas European countries exhibit stronger institutional trust and proactive help-seeking. Mexico lacks robust public long-term care frameworks, potentially shifting costs to informal caregivers rather than formal inpatient services, aligning with lower observed inpatient costs [45]. In Mexico, high income inequality may restrict outpatient access for lower-income groups despite cognitive needs [46], whereas European redistributive welfare systems mitigate such barriers.

Previous studies showed that cognitive frailty was an independent risk factor for hospitalization, and the risk value was higher than cognitive impairment alone or frailty alone [12, 13]. Our findings on inpatient services utilization in the KLoSA, MHAS and SHARE cohorts are consistent with previous findings. Furthermore, the coefficient between cognitive frailty and annual OOP costs in KLoSA and SHARE was also larger than that between frailty alone and annual OOP costs. Cognitive frailty may be a physiological precursor to the degenerative nervous system diseases, which could play a key role in predicting the short- and long-term adverse health outcomes such as falls, disability, dementia, and all-cause mortality [12, 13]. Participants with cognitive frailty had a higher dementia or mortality risk compared with those with only cognitive impairment status or only frailty status [9, 47]. These may all increase health care utilization or OOP costs among participants with cognitive frailty.

However, no such findings were found for outpatient service utilization in the four cohorts or inpatient service utilization in CHARLS. Low utilization or expense of participants with cognitive impairment could be due to several reasons: Firstly, underutilization of the health care services for participants with cognitive impairment. Secondly, those with cognitive frailty had lower economic levels (Table 1). It is worth mentioning that participants with higher income levels can spend more on health care services and gain more health [48]. Likewise, participants with lower income levels spend less on health care services and are expected to gain less health compared to others.

OOP costs reflect only a fraction of total healthcare expenditures, as they exclude public insurance payments and private coverage—components critical for assessing true burden. In cohorts with robust public healthcare (e.g., SHARE), OOP costs represent a small portion of total costs, meaning our findings likely underestimate cognitive frailty’s impact. For instance, long-term care and inpatient services for cognitive frailty, often subsidized in such systems, are undercounted. Conversely, in settings with limited coverage (e.g., CHARLS and MHAS), OOP better approximates total costs but still misses unmeasured indirect costs. We propose longitudinal studies integrating claims data, caregiver-reported indirect costs, and health system expenditure records to better quantify the full economic burden.

This study has some policy implications. If individuals with cognitive impairments are utilizing fewer healthcare resources, it might indicate a need for better outreach and support services to ensure they receive appropriate care. Understanding cognitive impairment and frailty can help healthcare providers allocate resources more effectively and develop targeted interventions. This could help in reducing the burden of health care costs on individuals and the healthcare system as a whole. In countries with more developed healthcare infrastructure, comprehensive geriatric assessment programs staffed by multidisciplinary teams could be feasible. In contrast, countries with limited resources might start with simpler screening tools and task-shifting to train primary care providers in recognizing early signs of cognitive impairment and frailty. In regions where frailty is more prevalent and linked to higher costs, allocating resources towards preventive frailty-reduction programs may be beneficial. Conversely, areas with higher rates of cognitive impairment might focus on developing specialized services for this population. We suggest future research should collaborate with policymakers and healthcare stakeholders at the country - specific level. This would help identify which integrated care models and payment reforms are most likely to succeed given local cultural, socioeconomic, and healthcare system factors.

There are several limitations in this study. First, due to data limitations, we relied on the memory and orientation domains to determine the presence of cognitive impairment. This may lack important cognitive domains (e.g., executive function). Future studies can consider a more comprehensive cognitive assessment that includes multiple domains to provide a fuller picture of cognitive health. Second, only OOP costs were available in this study, thus we could not include important cost variables such as global health care costs. OOP might represent only a minor portion of the total costs, potentially underestimating the overall impact. Third, cultural, socioeconomic, and healthcare system differences can influence the prevalence of cognitive frailty and healthcare utilization patterns. We are therefore unable to combine data from the four cohorts, only to observe their similar features. But comparing utilization and especially OOP costs may be biased by system-level differences (e.g., coverage, pricing, availability). It is necessary for caution when interpreting the results across different healthcare contexts. Finally, while many covariates are adjusted for, unmeasured factors (e.g., caregiving support, dementia awareness, health literacy) may still bias the association between cognitive frailty and healthcare use.

In conclusion, our study reveals nuanced associations between cognitive impairment, frailty, cognitive frailty and health care utilization and costs. We found that only cognitive impairment was associated with reduced health care utilization or annual OOP costs, while only frailty was linked to higher health care utilization or costs. Cognitive frailty was positively associated with and health care utilization and OOP costs in most countries. Future research should further explore these associations across diverse settings to enhance our understanding of cognitive frailty and its implications for healthcare systems.

## Supplementary Materials

The Supplementary data can be found online at: www.aginganddisease.org/EN/10.14336/AD.2025.0722.



## Data Availability

The data that support the findings of this study are available from the Gateway to Global Aging Data (https://g2aging.org/).
