# Supplementary material for: Cognitive Frailty and Health Care Utilization and Costs Among Middle-Aged and Older Adults: Findings from Four Longitudinal Cohort Studies
Source: Aging Dis. 2026 Jul 31;17(5):2740–52. doi: 10.14336/AD.2025.0722 (PMC13437102; doi:10.14336/AD.2025.0722)
Supplement: Supplementary file 1 [file AD-17-5-2740-s.pdf]

SUPPLEMENTARY DATA

Cognitive Frailty and Health Care Utilization and Costs Among Middle-Aged and Older Adults: Findings from Four Longitudinal Cohort Studies

Yemin Yuan, Zhenyu Shi, Xiaolong Guan, Yiqi Xia, Yanshang Wang, Huaxin Si, Ping He

© 2025. Yuan Y et al. Published online at http://www.aginganddisease.org/EN/10.14336/AD.2025.0722

SUPPLEMENTARY DATA

Contents:

Supplementary Table 1. Wave and time in CHARLS, KLoSA, MHAS, and SHARE. 3
Supplementary Figure 1. Flowchart of sample selection..3
Supplementary Table 2. Three sets of cognitive function tests. 4
Supplementary Table 3. The 29 items used to construct the frailty index. 5
Supplementary Table 4. Description of health care utilization and costs in the four cohorts. 7
Supplementary Methods 8
Supplementary Table 5. Distribution of health care use. 9
Supplementary Table 6. The newly added items used to reconstruct the frailty index....................................10
Supplementary Table 7. Regression results of cognitive frailty on health care utilization and total aggregated annual out-of-pocket costs. 11
Supplementary Table 8. Regression results of cognitive frailty on health care utilization and costs based on inverse probability-weighted samples. 12
Supplementary Table 9. Two-part models of cognitive frailty on health care utilization and costs. 13
Supplementary Table 10. Regression results of cognitive frailty on health care utilization and costs after the newly constructed frailty index. 14

© 2025. Yuan Y et al. Published online at http://www.aginganddisease.org/EN/10.14336/AD.2025.0722

SUPPLEMENTARY DATA

Supplementary Table 1. Wave and time in CHARLS, KLoSA, MHAS, and SHARE.

	**CHARLS**	**KLoSA**	**MHAS**	**SHARE**
Wave: date	wave1: 2011	wave3: 2010	wave3: 2012	wave5: 2013
	wave2: 2013	wave4: 2012	wave4: 2015	wave6: 2015
	wave3: 2015	wave5: 2014	wave5: 2018	wave7: 2017
	wave4: 2018	wave6: 2016		
		wave7: 2018		

CHARLS: China Health and Retirement Longitudinal Study; KLoSA: Korean Longitudinal Study of Aging; MHAS: Mexican Health and Aging Study; SHARE: Survey of Health, Ageing and Retirement in Europe.

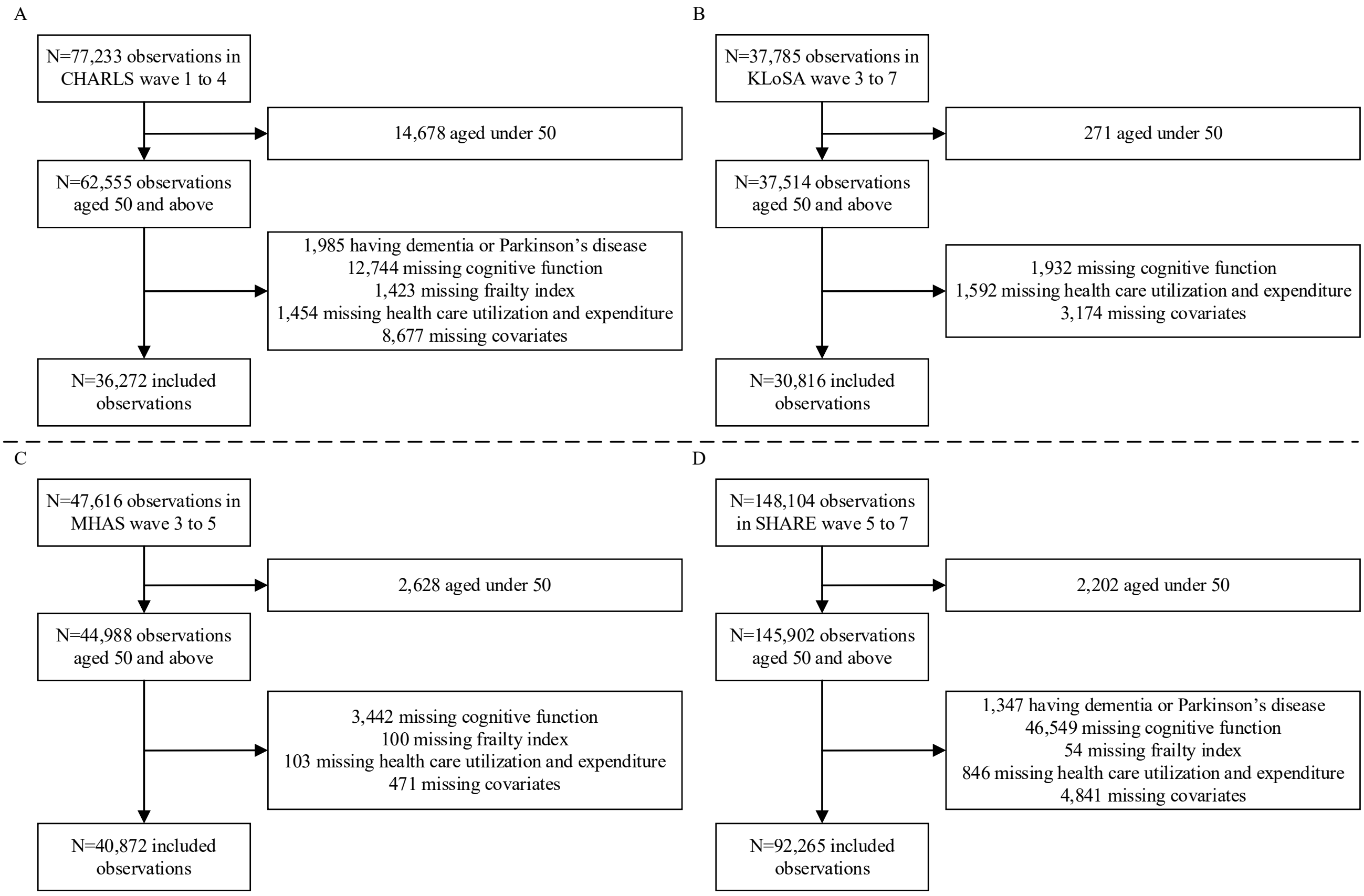

Supplementary Figure 1. Flowchart of sample selection.
CHARLS: China Health and Retirement Longitudinal Study; KLoSA: Korean Longitudinal Study of Aging; MHAS: Mexican Health and Aging Study; SHARE: Survey of Health, Ageing and Retirement in Europe.

SUPPLEMENTARY DATA

Supplementary Table 2. Three sets of cognitive function tests.

Items	Scoring range			
	**CHARLS**	**KLoSA**	**MHAS**	**SHARE**
Immediate word recall	0-10	0-3	0-8	0-10
Delayed word recall	0-10	0-3	0-8	0-10
Total recall summary score	0-20	0-6	0-16	0-20
Date naming-day of week	0-1	0-1	-	0-1
Date naming-day of month	0-1		0-1	0-1
Date naming-month	0-1	0-3	0-1	0-1
Date naming-year	0-1		0-1	0-1
Orientation (summary date naming)	0-4	0-4	0-3	0-4

For the immediate and delayed word recall, participants were asked to recite 10 words in CHARLS and SHARE; 3 words in KLoSA; and 8 words in MHAS. Participants were asked if they could remember the date of that day — specifically, day of week, day of month, month, and year in CHARLS, KLoSA, and SHARE; day of month, month, and year in MHAS — to measure the orientation to the date.

CHARLS: China Health and Retirement Longitudinal Study; KLoSA: Korean Longitudinal Study of Aging; MHAS: Mexican Health and Aging Study; SHARE: Survey of Health, Ageing and Retirement in Europe.

Supplementary Table 3. The 29 items used to construct the frailty index.

No	Description of the items				Cut-off value
	**CHARLS**	**KLoSA**	**MHAS**	**SHARE**	
1	Self-reported health				Excellent=0, Very good=0.25, Good=0.50, Fair =0.75, Poor=1.00
2	Some difficulty with dressing	Need for help with dressing	Some difficulty with dressing		Yes=1.00, No=0.00
3	Some difficulty with bathing or showering	Need for help with bathing	Some difficulty with bathing or showering		Yes=1.00, No=0.00
4	Some difficulty with eating	Need for help with eating	Some difficulty with eating		Yes=1.00, No=0.00
5	Some difficulty with getting in and out of bed	Need for help with getting in and out of bed and cross room	Some difficulty with getting in and out of bed		Yes=1.00, No=0.00
6	Some difficulty with using the toilet	Need for help with using the toilet	Some difficulty with using the toilet		Yes=1.00, No=0.00
7	Some difficulty with managing money	Need for help with managing money	Some difficulty with managing money		Yes=1.00, No=0.00
8	Some difficulty with taking medications	Need for help with taking medications	Some difficulty with taking medications		Yes=1.00, No=0.00
9	Some difficulty with shopping for groceries	Need for help with shopping	Some difficulty with shopping for groceries		Yes=1.00, No=0.00
10	Some difficulty with preparing hot meals	Need for help with preparing hot meals	Some difficulty with preparing hot meals		Yes=1.00, No=0.00
11	Some difficulty with walking 100 meters	Need for help with brushing teeth	Some difficulty with 100 meters/1 block		Yes=1.00, No=0.00
12	Some difficulty with getting up from a chair after sitting for long periods	Need for help with controlling urination and defecation	Some difficulty with getting up from a chair after sitting for long periods		Yes=1.00, No=0.00
13	Some difficulty with climbing several flights of stairs without resting	Need for help with using transportation	Some difficulty with climbing several flights of stairs without resting		Yes=1.00, No=0.00
14	Some difficulty with stooping, kneeling, or crouching	Need for help with going out short distance without using transportation	Some difficulty with stooping, kneeling, or crouching		Yes=1.00, No=0.00
15	Some difficulty with lifting or carrying weights over 10 jins	Need for help with doing laundry	Some difficulty with lifting or carrying weights over 10 pounds/ 5 kilograms		Yes=1.00, No=0.00
16	Some difficulty with picking up a coin from the table	Need for help with doing housework	Some difficulty with picking up a small coin from the table		Yes=1.00, No=0.00
17	Some difficulty with reaching arms above shoulder level	Need for help with personal grooming	Some difficulty with reaching arms above shoulder level		Yes=1.00, No=0.00

18	Self-reported physician diagnosed hypertension	Yes=1.00, No=0.00
19	Self-reported physician diagnosed diabetes	Yes=1.00, No=0.00
20	Self-reported physician diagnosed cancer	Yes=1.00, No=0.00
21	Self-reported physician diagnosed chronic lung disease	Yes=1.00, No=0.00
22	Self-reported physician diagnosed heart disease	Yes=1.00, No=0.00
23	Self-reported physician diagnosed stroke	Yes=1.00, No=0.00
24	Self-reported physician diagnosed arthritis	Yes=1.00, No=0.00
25	Feeling depressed most of time	Yes=1.00, No=0.00
26	Feeling that everything is an effort most of time	Yes=1.00, No=0.00
27	Feeling that sleep was restless most of time	Yes=1.00, No=0.00
28	Feeling happy most of time	No=1.00, Yes=0.00
29	Feeling enjoying life most of time	No=1.00, Yes=0.00

To harmonize the frailty assessment of data from the four cohorts, we selected 29 items to construct the frailty index (FI), including variables of self-reported health status, functional limitations, mobility, chronic diseases, and psychological characteristics. We excluded participants that they had >20% missing data in items of frailty index. FI was calculated as the sum of scoring divided by the total number of items.

CHARLS: China Health and Retirement Longitudinal Study; KLoSA: Korean Longitudinal Study of Aging; MHAS: Mexican Health and Aging Study; SHARE: Survey of Health, Ageing and Retirement in Europe.

SUPPLEMENTARY DATA

Supplementary Table 4. Description of health care utilization and costs in the four cohorts.

	CHARLS	KLoSA	MHAS	SHARE
**Utilization**				
Outpatient	Whether the respondent reports they have, in the past month, visited a public hospital, private hospital, public health center, clinic, or health worker's or doctor's practice, or been visited by a health worker or doctor for outpatient care.	Whether the respondent has visited a public health clinic or seen or talked to a medical doctor in the past two years.	Whether the respondent reports at least one doctor visit in the last 12 months.	Whether the respondent reports any doctor visit in the last 12 months. For doctor visits, the question asks how many times the respondent has seen or talked to a medical doctor including emergency room or outpatient clinic visits.
Outpatient, number	The number of visits to any of the below medical facilities that the respondents had during last month for outpatient treatment.	The number of times that the respondent has visited a public health clinic or has seen a doctor in the past two years.	The reported number of visits in the last 12 months.	The reported number of doctor visits in the last 12 months.
Inpatient	Whether the respondent reports receiving any inpatient care in the past year.	Whether the respondent has been hospitalized at a hospital, nursing home, convalescent home or other long-term health care facility in the past two years.	Whether the respondent reports at least one overnight hospital stay in the last 12 months.	Whether the respondent reports any overnight hospital stay in the last 12 months.
Inpatient, number	The number of times that the respondent has received inpatient care in the past year.	The number of times that the respondent has been hospitalized at the hospital, nursing home, convalescent home or other long-term health care facility in the past two years.	/	The reported number of hospital stays from 1 to 10 in the last 12 months.
**Costs**				
Outpatient costs, out-of-pocket	The value of out-of-pocket doctor visit expenditures for the respondent in the last month, including the fees paid for treatment, medication costs and prescription drugs.	The value of out of pocket expenditures for visits to the doctor and an emergency room in the past two years.	The total out of pocket doctor visit expenditures in the last 12 months.	The amount the respondent reported spending out-of-pocket on outpatient care in the previous year.
Inpatient costs, out-of-pocket	The value of out-of-pocket hospitalization expenditure for the respondent in the past year, including the fees paid to the hospital but excluding the wages paid to a hired nurse, transportation costs, and accommodation costs for respondent and family members.	The sum of out of pocket expenditures for hospitalization (medical treatment) and hospital caregiver services costs in the past two years.	The out of pocket hospitalizations expenditure in the last 12 months.	The amount the respondent reported spending out-of-pocket on hospitalizations in the previous year.

CHARLS: China Health and Retirement Longitudinal Study; KLoSA: Korean Longitudinal Study of Aging; MHAS: Mexican Health and Aging Study; SHARE: Survey of Health, Ageing and Retirement in Europe.

SUPPLEMENTARY DATA

Supplementary Methods

The sex was reported as either male or female. According to the International Standard Classification of Education (ISCED) 1997, education is classified as less than upper secondary, upper secondary and vocational training, and tertiary. Residence refers to whether the participants live in an urban or rural area. The level of household wealth has been divided into tertiles of non-housing financial wealth. The household wealth in CHARLS, KLoSA and SHARE is at the couple level, while the household wealth in MHAS is at the household level. Marital status was categorised as married/partnered or single, single covered separated, divorced, widowed, and never married. Smoking was described as current smoking behavior or smoking in the past. Drinking was about whether alcohol was consumed last week, last three months, last year, or earlier. Public health insurance indicates whether the respondent is covered by any public (government) health insurance program. The health insurance variable is only available in wave 1 in SHARE, and there are many missing values.

The Heckman sample selection models and the two-part models are the two main models for handling healthcare costs data with a large number of zero values [1]. The similarities between the two models are as follows [2,3]: the first part is about whether an individual chooses to seek medical treatment, and the second part is about the medical expenditures after seeking medical treatment. The differences between the two models are as follows: first, in the two-part model, there is no correlation between the decision of whether to seek medical treatment in the first part and the medical expenditures in the second part. Mechanisms of the two decision-making processes are different. In the sample selection model, the decision of whether to seek medical treatment in the first part is affected by the potential level of medical expenditures in the second part. After getting sick, an individual faces a decision-making process of whether to seek medical treatment, and there is a close relationship between the decision of whether to seek medical treatment and the medical expenditures. The selection model can correct the self-selection bias in the costs model. Second, the two models handle medical expenditures with zero-value differently. The two-part model treats zero-value medical expenditures as real values, while the sample selection model believes that zero-value medical expenditures may not be the actual medical expense expenditures but are caused by selection bias. Considering the certain differences in the assumptions of the two models, this study adopted both the sample selection model and the two-part model (using the latter as a sensitivity analysis). By comparing the estimation results of the two models, we ensured the robustness of the estimation results.

We used the Box-Cox approach and the modified Park test to determine the link function and distribution of the generalized linear model (GLM) [4]. Test for the link function: a power of $\delta = 1$ corresponds to a linear model, $\delta = 0.5$ corresponds to the square root transformation, and $\delta = 0$ corresponds to the natural log transformation model. The estimated coefficients (/theta in the output) for outpatient and inpatient costs are closer to zero (CHARLS: -.006, 0.016; KLoSA: -0.128, 0.065; MHAS: 0.032, 0.189; SHARE: 0.047, -0.021). We take this to mean that the log link function is preferable to the square root or other common link functions. Test for the distribution family: the Park test is based on the estimated relationship between the variance of the error term and mean. The dependent variable is the natural logarithm of the raw-scale variance. The sole covariate is the natural log of the conditional expected value of the dependent variable, $x_i'\hat{\beta} = \ln\{E(y_i \mid x_i)\}$. The coefficient on the predicted value indicates which distribution family is preferred. Typically, analysts have made choices that reflect integer powers: If it is close to 0, use the Gaussian family, because the variance is unrelated to the mean; If it is close to 1, use the Poisson, because of its property that the variance is proportional to the mean; If it is close to 2, use the gamma; If it is close to three, use the inverse Gaussian. The estimated coefficient for outpatient and inpatient costs on xbetahat (CHARLS: 1.868, 1.896; KLoSA: 1.732, 2.019; MHAS: 1.866, 2.045; SHARE: 1.501, 2.057) is closest to the gamma family's integer value of 2. Therefore, for these data and this model specification, we choose the gamma family.

References

1. Leung SF, Yu S. On the choice between sample selection and two-part models. J Econom. 1996;72:197-229.

2. Duan N, Newhouse JP, Morris CN *et al*. A Comparison of Alternative Models for the Demand for Medical Care. J Bus Econ Stat, 1983;1:115-26.

3. Cameron AC, Trivedi PK. Microeconometrics: Methods and applications. Cambridge, Cambridge University Press. 2005.

4. Partha D & Edward CN, Willard GM. Health Econometrics Using Stata. College Station, Stata Press. 2017.

SUPPLEMENTARY DATA

Supplementary Table 5. Distribution of health care use.

	Number of outpatient visits			Number of inpatient admissions		
	Mean	Variance	n (zero), %	Mean	Variance	n (zero), %
CHARLS (n=36,272)	4.665	250.234	30,057 (82.9)	0.192	0.415	31,613 (87.2)
KLoSA (n=30,816)	6.855	116.605	5,637 (18.3)	0.073	0.077	27,313 (88.6)
MHAS (n=40,872)	5.739	58.395	10,106 (24.7)	-	-	-
SHARE (n=92,265)	6.629	85.425	6,139 (6.7)	0.259	0.771	78,351 (84.9)

SUPPLEMENTARY DATA

Supplementary Table 6. The newly added items used to reconstruct the frailty index.

No	Description of the items				Cut-off value
	CHARLS	KLoSA	MHAS	SHARE	
30	Some difficulty with controlling urination and defecation	Visual impairment (close)	Change in self-rated health	Visual impairment (close)	Yes=1.00, No=0.00
31	Some difficulty with cleaning house	Visual impairment (distant)	Hearing impairment	Visual impairment (distant)	Yes=1.00, No=0.00
32	Some difficulty with running or jogging 1 kilometer	Hearing impairment	Pain	Hearing impairment	Yes=1.00, No=0.00
33	Self-reported physician diagnosed psychiatric problem	Pain	Body mass index deficit	Pain	Yes=1.00, No=0.00
34	Self-reported physician diagnosed dyslipidemia	Body mass index deficit	Some difficulty with walking across room	Body mass index deficit	Yes=1.00, No=0.00
35	Self-reported physician diagnosed liver disease	Need for help with using telephone	Some difficulty with walking several blocks	Some difficulty with walking across room	Yes=1.00, No=0.00
36	Self-reported physician diagnosed kidney disease	Self-reported physician diagnosed psychiatric problem	Some difficulty with jogging 1 kilometer	Some difficulty with doing housework	Yes=1.00, No=0.00
37	Self-reported physician diagnosed stomach or other digestive disease	Self-reported physician diagnosed liver disease	Some difficulty with sitting for two hours	Some difficulty with using telephone	Yes=1.00, No=0.00
38	Self-reported physician diagnosed asthma	Felt lonely most of time	Some difficulty with climbing one flight of stairs	Some difficulty with using a map	Yes=1.00, No=0.00
39	Feeling lonely most of time	Could not get going most of time	Some difficulty with pulling or pushing large objects	Some difficulty with sitting for two hours	Yes=1.00, No=0.00
40	Bothered by little things most of time	-	Feeling tired most of time	Some difficulty with climbing one flight of stairs	Yes=1.00, No=0.00
41	Could not get going most of time	-	Feeling lonely most of time	Some difficulty with pulling or pushing large objects	Yes=1.00, No=0.00
42	Having trouble keeping mind most of time	-	Feeling sad most of time	Self-reported physician diagnosed psychiatric problem	Yes=1.00, No=0.00
43	Feeling fearful most of time	-	Having a lot of energy most of time (reverse-coded)	Having difficulty in concentrating	Yes=1.00, No=0.00

On the basis of the 29 items, the entries in the following table were added to each cohort. New frailty indexes of 43 (CHARLS, MHAS, and SHARE) and 39 (KLoSA) items were constructed respectively.

CHARLS: China Health and Retirement Longitudinal Study; KLoSA: Korean Longitudinal Study of Aging; MHAS: Mexican Health and Aging Study; SHARE: Survey of Health, Ageing and Retirement in Europe.

SUPPLEMENTARY DATA

Supplementary Table 7. Regression results of cognitive frailty on health care utilization and total aggregated annual out-of-pocket costs.

	**CHARLS (n=36,272)**		**KLoSA (n=30,816)**	
	Selection model, whether it occurs	Costs Model, ln (costs)	Selection model, whether it occurs	Costs Model, ln (costs)
Outpatient + Inpatient				
None	Ref	Ref	Ref	Ref
Only cognitive impairment	-0.088 (0.056)	-0.162 (0.085)	-0.211** (0.064)	-0.043 (0.031)
Only frailty	1.044*** (0.037)	0.596*** (0.047)	0.558*** (0.141)	0.677*** (0.053)
Cognitive frailty	0.849*** (0.064)	0.497*** (0.083)	1.144 (0.128)	0.570*** (0.058)
	**MHAS (n=40,872)**		**SHARE[a] (n=92,265)**	
	Selection model, whether it occurs	Costs Model, ln (costs)	Selection model, whether it occurs	Costs Model, ln (costs)
Outpatient + Inpatient				
None	Ref	Ref	Ref	Ref
Only cognitive impairment	-0.042*** (0.008)	-0.091(0.068)	-0.512*** (0.070)	-0.002 (0.029)
Only frailty	0.128*** (0.005)	0.554*** (0.040)	1.877*** (0.075)	0.086*** (0.016)
Cognitive frailty	0.092*** (0.009)	0.412*** (0.070)	0.579*** (0.096)	0.126*** (0.033)

CHARLS: China Health and Retirement Longitudinal Study; KLoSA: Korean Longitudinal Study of Aging; MHAS: Mexican Health and Aging Study; SHARE: Survey of Health, Ageing and Retirement in Europe.

Models were adjusted for: age, sex, education, residency, household wealth, marital status, smoking, alcohol drinking, public health insurance, and survey year. Standard errors in parentheses.

[a]SHARE was not adjusted for insurance variables. ****P*<0.001, ***P*<0.01, **P*<0.05.

SUPPLEMENTARY DATA

Supplementary Table 8. Regression results of cognitive frailty on health care utilization and costs based on inverse probability-weighted samples.

	**CHARLS (n=36,272)**		**KLoSA (n=30,816)**	
	Selection model, whether it occurs	Costs Model, ln (costs)	Selection model, whether it occurs	Costs Model, ln (costs)
Outpatient				
None	Ref	Ref	Ref	Ref
Only cognitive impairment	-0.097 (0.074)	-0.170* (0.084)	-0.270*** (0.074)	-0.060* (0.027)
Only frailty	0.838*** (0.048)	0.484*** (0.047)	0.022 (0.135)	0.438*** (0.059)
Cognitive frailty	0.742*** (0.077)	0.358*** (0.082)	-0.434** (0.131)	0.260*** (0.055)
Inpatient				
None	Ref	Ref	Ref	Ref
Only cognitive impairment	-0.077 (0.085)	-0.069 (0.096)	-0.016 (0.082)	-0.064 (0.104)
Only frailty	1.185*** (0.052)	0.177*** (0.047)	1.298*** (0.105)	0.382** (0.127)
Cognitive frailty	0.972*** (0.084)	0.117 (0.082)	1.292*** (0.110)	0.680*** (0.133)
	**MHAS (n=40,872)**		**SHARE[a] (n=92,265)**	
	Selection model, whether it occurs	Costs Model, ln (costs)	Selection model, whether it occurs	Costs Model, ln (costs)
Outpatient				
None	Ref	Ref	Ref	Ref
Only cognitive impairment	-0.141 (0.103)	-0.095 (0.063)	-0.590*** (0.140)	0.010 (0.030)
Only frailty	0.927*** (0.099)	0.451*** (0.037)	1.881*** (0.146)	0.025 (0.017)
Cognitive frailty	0.725*** (0.118)	0.313*** (0.066)	0.842*** (0.182)	0.058 (0.034)
Inpatient				
None	Ref	Ref	Ref	Ref
Only cognitive impairment	-0.068 (0.134)	-0.412* (0.191)	0.073 (0.101)	-0.104 (0.096)
Only frailty	1.212*** (0.091)	-0.109 (0.104)	1.283*** (0.057)	0.117** (0.044)
Cognitive frailty	1.367*** (0.123)	-0.456** (0.165)	1.438*** (0.087)	0.207** (0.077)

CHARLS: China Health and Retirement Longitudinal Study; KLoSA: Korean Longitudinal Study of Aging; MHAS: Mexican Health and Aging Study; SHARE: Survey of Health, Ageing and Retirement in Europe.

Models were adjusted for: age, sex, education, residency, household wealth, marital status, smoking, alcohol drinking, public health insurance, and survey year. Standard errors in parentheses.

[a]SHARE was not adjusted for insurance variables. ***P<0.001, **P<0.01, *P<0.05.

SUPPLEMENTARY DATA

Supplementary Table 9. Two-part models of cognitive frailty on health care utilization and costs.

	**CHARLS (n=36,272)**		**KLoSA (n=30,816)**	
	Part I (logit model)	Part II (GLM model)	Part I (logit model)	Part II (GLM model)
Outpatient				
None	Ref	Ref	Ref	Ref
Only cognitive impairment	-0.082 (0.057)	-0.060 (0.165)	-0.255*** (0.043)	-0.051 (0.078)
Only frailty	0.775*** (0.034)	0.605*** (0.091)	-0.510*** (0.069)	0.726*** (0.135)
Cognitive frailty	0.667*** (0.060)	0.309 (0.165)	-1.046*** (0.066)	0.359* (0.147)
Inpatient				
None	Ref	Ref	Ref	Ref
Only cognitive impairment	-0.073 (0.069)	-0.033 (0.128)	0.028 (0.087)	-0.003 (0.125)
Only frailty	1.044*** (0.038)	0.211** (0.067)	0.875*** (0.105)	0.560*** (0.142)
Cognitive frailty	0.876*** (0.066)	0.207 (0.114)	0.676*** (0.117)	0.672*** (0.157)
	**MHAS (n=40,872)**		**SHARE[a] (n=92,265)**	
	Part I (logit model)	Part II (GLM model)	Part I (logit model)	Part II (GLM model)
Outpatient				
None	Ref	Ref	Ref	Ref
Only cognitive impairment	-0.175*** (0.047)	-0.037 (0.097)	-0.411*** (0.031)	0.029 (0.039)
Only frailty	0.239*** (0.029)	0.503***(0.058)	0.257*** (0.019)	-0.006 (0.021)
Cognitive frailty	0.227*** (0.050)	0.397*** (0.099)	-0.340*** (0.034)	-0.031 (0.044)
Inpatient				
None	Ref	Ref	Ref	Ref
Only cognitive impairment	0.060 (0.111)	-0.176 (0.157)	-0.031 (0.071)	-0.301 (0.194)
Only frailty	0.801*** (0.064)	0.099 (0.088)	1.112*** (0.033)	-0.107 (0.089)
Cognitive frailty	0.858*** (0.095)	-0.133 (0.134)	0.816*** (0.059)	0.058 (0.156)

CHARLS: China Health and Retirement Longitudinal Study; KLoSA: Korean Longitudinal Study of Aging; MHAS: Mexican Health and Aging Study; SHARE: Survey of Health, Ageing and Retirement in Europe; GLM: generalized linear model.

Models were adjusted for: age, sex, education, residency, household wealth, marital status, smoking, alcohol drinking, public health insurance, and survey year. Standard errors in parentheses.

[a]SHARE was not adjusted for insurance variables. ***P<0.001, **P<0.01, *P<0.05.

SUPPLEMENTARY DATA

Supplementary Table 10. Regression results of cognitive frailty on health care utilization and costs after the newly constructed frailty index.

	**CHARLS (n=36,186)**		**KLoSA (n=30,811)**	
	Selection model, whether it occurs	Costs Model, ln (costs)	Selection model, whether it occurs	Costs Model, ln (costs)
Outpatient				
None	Ref	Ref	Ref	Ref
Only cognitive impairment	-0.088 (0.062)	-0.148 (0.079)	-0.207** (0.063)	-0.062* (0.027)
Only frailty	0.908*** (0.044)	0.501*** (0.048)	0.257* (0.107)	0.437*** (0.039)
Cognitive frailty	0.757*** (0.076)	0.332*** (0.086)	-0.298** (0.110)	0.249*** (0.045)
Inpatient				
None	Ref	Ref	Ref	Ref
Only cognitive impairment	-0.050 (0.071)	-0.141 (0.084)	0.007 (0.074)	-0.086 (0.106)
Only frailty	1.344*** (0.048)	0.191*** (0.048)	1.294*** (0.081)	0.231* (0.102)
Cognitive frailty	1.016*** (0.081)	0.186* (0.083)	1.241*** (0.089)	0.579*** (0.127)
	**MHAS (n=40,845)**		**SHARE[a] (n=92,279)**	
	Selection model, whether it occurs	Costs Model, ln (costs)	Selection model, whether it occurs	Costs Model, ln (costs)
Outpatient				
None	Ref	Ref	Ref	Ref
Only cognitive impairment	-0.214*** (0.059)	-0.123 (0.068)	-0.350*** (0.063)	0.027 (0.030)
Only frailty	0.998*** (0.039)	0.410*** (0.033)	1.994*** (0.064)	0.036* (0.016)
Cognitive frailty	0.684*** (0.066)	0.316*** (0.058)	0.805*** (0.087)	0.042 (0.033)
Inpatient				
None	Ref	Ref	Ref	Ref
Only cognitive impairment	0.078 (0.088)	-0.249 (0.222)	0.084 (0.052)	-0.147 (0.095)
Only frailty	1.210*** (0.043)	-0.151 (0.108)	1.304*** (0.029)	0.137** (0.044)
Cognitive frailty	1.319*** (0.065)	-0.578*** (0.154)	1.347*** (0.046)	0.247** (0.076)

CHARLS: China Health and Retirement Longitudinal Study; KLoSA: Korean Longitudinal Study of Aging; MHAS: Mexican Health and Aging Study; SHARE: Survey of Health, Ageing and Retirement in Europe.

Models were adjusted for: age, sex, education, residence, household wealth, marital status, smoking, alcohol drinking, public health insurance, and survey year. Standard errors in parentheses.

[a]SHARE was not adjusted for insurance variables. ****P*<0.001, ***P*<0.01, **P*<0.05.
